# Successful Integration of Membrane Technologies in a Conventional Purification Process of Tannery Wastewater Streams

**DOI:** 10.3390/membranes3030126

**Published:** 2013-07-11

**Authors:** Marco Stoller, Olga Sacco, Diana Sannino, Angelo Chianese

**Affiliations:** 1 Department of Chemical Engineering, University of Rome “La Sapienza”, Via Eudossiana 18, 00184 Rome, Italy; E-Mail: angelo.chianese@uniroma1.it; 2 Department of Chemical Engineering, University of Salerno, Via Ponte Don Melillo, 84084 Fisciano, Italy; E-Mails: osacco@unisa.it (O.S.); dsannino@unisa.it (D.S.)

**Keywords:** threshold flux, critical flux, nanofiltration, tannery wastewater, process optimization

## Abstract

The aim of this work is to design and integrate an optimized batch membrane process in a conventional purification process used for the treatment of tannery wastewater. The integration was performed by using two spiral wound membrane modules in series, that is, nanofiltration and reverse osmosis, as substitutes to the biological reactor. The membrane process was designed in terms of sensible fouling issues reduction, which may be observed on the nanofiltration membrane if no optimization is performed. The entity of the fouling phenomena was estimated by pressure cycling measurements, determining both the critical and the threshold flux on the nanofiltration membrane. The obtained results were used to estimate the need of the overdesign of the membrane plant, as well as to define optimized operating conditions in order to handle fouling issues correctly for a long period of time. Finally, the developed membrane process was compared, from a technical and economic point of view, with the conventional biological process, widely offered as an external service near tannery production sites, and, here, proposed to be substituted by membrane technologies.

## 1. Introduction

The treatment and purification of tannery wastewater is a difficult task to accomplish, due to the variety and toxicity of the chemicals added during the different stages of the production of hides and skins. The wastewater contains, among others, sensible amounts of heavy metals, toxic chemicals, chloride, lime and highly dissolved and suspended salts, representing an environmental threat if not properly treated before discharge [1]. Throughout the years, research focused on many different conventional treatment processes of industrial wastewater effluents, such as biological process, oxidation process and chemical process [2,3,4,5,6,7,8,9,10,11,12,13,14,15,16,17,18]. Unfortunately, in the case of tannery wastewater streams, biological treatment methods give rise to an excessive production of sludge, whereas physical and chemical methods are too expensive in terms of energy and reagent costs [19,20,21]. Therefore, the treatment of this wastewater needs technical reorganization, by combining and integrating alternative systems to the conventional ones. 

In this work, the possibility to integrate membrane processes is checked. In particular, two batch membrane processes in series, that is, nanofiltration (NF) and reverse osmosis (RO), were used in order to completely avoid the need to rely on external treatment services, capable of purifying this wastewater by conventional biological treatment steps. 

One main problem in membrane technologies is membrane fouling, which sensibly affects the process performances in terms of productivity and longevity. Field *et al.* introduced in 1995 the concept of critical flux for microfiltration, stating that there is a permeate flux below which fouling is not promptly observed, and the validity of this concept was later on confirmed for ultrafiltration and nanofiltration membranes, too [22]. On the other hand, in the case of the treatment of real waste water streams, Le Clech *et al.* noticed that operations below the critical flux may not be sufficient in order to have zero fouling rates [23]. Therefore, it appears that membrane systems treating real wastewater streams do not exhibit a critical flux in a strict way. To overcome this limitation in the definition of critical flux, in a recent paper, in the year 2011, Field and Pearce introduced for the first time the concept of threshold flux [24]. Summarizing the concept briefly, the threshold flux is the flux that divides a low fouling region, characterized by a nearly constant rate of fouling, from a high fouling region, where flux-dependent high fouling rates can be observed. 

Critical and threshold fluxes were measured by adopting the pressure cycle protocol [25]. Once the critical and threshold flux values were obtained, this data was used as input for a batch membrane process optimization method developed previously by Stoller *et al.* [26,27,28,29,30,31,32,33,34,35]. 

## 2. Experimental setup

### 2.1. The Tannery Wastewater Stream

Tannery wastewater streams are characterized by a basic pH, dark brown color and have a high content of organic and suspended matter, reported in detail in Table 1.

The raw wastewater stream is processed by primary treatment processes listed here:

- Coarse gridding
- Sedimentation of sand
- Oil skimming
- Oxidation of sulfides
- Flocculation
- Flotation

After pretreatment, the tannery wastewater stream exhibits different characteristics. Even after the primary treatment processes, the characteristics are not compatible with discharging in surface water, and, therefore, need further purification. Details are reported in Table 2. 

**Table 1:** Characteristics of the raw tannery wastewater stream.

Parameter	ID	Unit	Value
Chemical oxygen demand	COD	mg/L	55,000
Total suspended solids	TSS	mg/L	985
Ammonium	NH_4_	mg/L	74
Phosphates	P	mg/L	2.6
Sulfides	S	mg/L	0.14
Chromium	Cr	mg/L	198

**Table 2:** Characteristics of the tannery wastewater stream after primary treatment processes.

Parameter	Unit	Value	Discharge limits
COD	mg/L	2,020	160
TSS	mg/L	266	80
NH_4_	mg/L	69	15
P	mg/L	2.5	10
S	mg/L	0.09	1
Cr	mg/L	195	2

Moreover, the effluents continue to be rich in nitrogen and poor in phosphorus, and the presence of sulfides and chromium exhibits high antibacterial activity. This situation makes bioremediation very difficult for reaching the desired purification targets. 

### 2.2. The Membrane Pilot Plant Used

**Figure 1:**
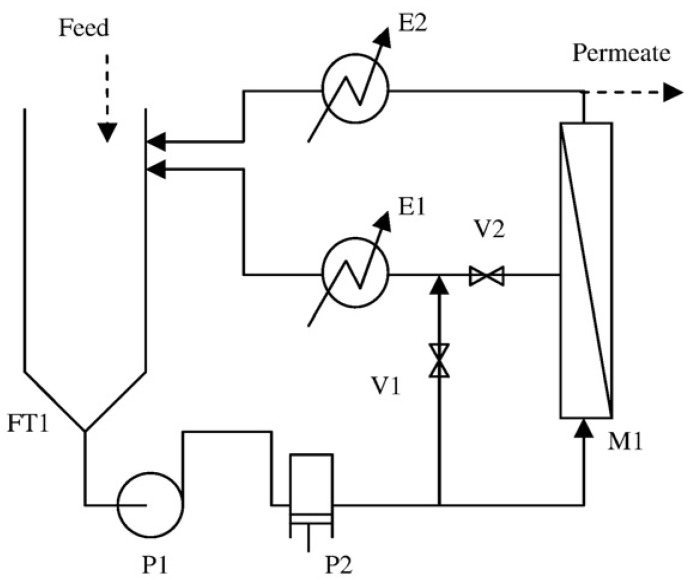
Scheme of the used membrane pilot plant.

The pilot plant used is shown schematically in Figure 1.

The plant consists of a 100 liter feed tank, FT1, in which the pretreated feedstock is carried. The centrifugal booster pump, P1, and the volumetric pump, P2, drive the wastewater stream over the used spiral wounded nanofiltration (NF model DK supplied by Osmonics) or reverse osmosis (RO model SC supplied by Osmonics) membrane, fitted in the housing, M1, at an average flow rate equal to 600 L h^−1^. The active membrane area of both the modules are equal to 0.51 m^2^. The maximum allowable operating pressure is equal to 32 bar and 64 bar for NF and RO, respectively.

Acting on the regulation valves, V1 and V2, it is possible to set the desired operating pressure over the membrane with a precision of 0.5 bar, maintaining the feed flow rate constant.

Permeate and concentrate streams are cooled down to the fixed feedstock temperature, mixed together and recycled back to the feedstock. In this way, the feedstock composition is kept constant during each experimental batch run. The temperature was controlled for all experiments at the value of 20 ± 1 °C.

After each experiment, the membrane was rinsed with tap water for at least 30 min. 

## 3. Results and Discussion

The first objective of this work was to identify optimal operating conditions for the two different membrane modules. 

The critical and threshold flux were measured for the NF membrane only, since RO did not show significant fouling issues in the adopted pressure range. The measurements were performed by applying the pressure cycle step method and successive evaluation method, described in detail elsewhere, starting from a value of 2 bar [25,32,33]. 

Concerning the critical flux, *J*c, the following fitting equations apply [22]:

−d*m*/d*t* = 0   *J*p(t) ≤ *J*c (1)

−d*m*/d*t* = B[*J*p(t) − *J*c]   *J*p(t) > *J*c (2)

where m is the permeability of the membrane and B is a fitting parameter.

The fitting equations proposed by Field *et al.* for the threshold flux *J*th are [24]:

−d*m*/d*t* = a   *J*p(t) ≤ *J*th (3)

−d*m*/d*t* = a + b[*J*p(t) − *J*th]   *J*p(t) > *J*th (4)

with a,b fitting parameters. 

Equations (1) and (2) are equal to Equations (3) and (4), if a = 0, b = B and *J*th is substituted by *J*c. Below critical flux, no fouling is observed; therefore, a constant contribution to the fouling phenomena is absent (a = 0). This is not the case below the threshold flux value, where fouling is immediately observed (a ≠ 0). Above critical and threshold flux values, the fouling behavior sensibly increases, and in both cases, fouling quickly starts to occur (b ≠ 0). 

Equations (1) and (3) can be discretized between *t*1 and *t*2, equal to one pressure cycling period, ∆*t*, and the following linear equation, hereafter marked by an asterisk, can be derived for the critical and threshold flux, respectively:

(−∆*m*/∆*t*)* = 0   *J*p(t) ≤ *J*c (5)

(−∆*m*/∆*t*)* = a   *J*p(t) ≤ *J*th (6)

As long as the adopted trans-membrane pressure (TMP) values remain below the threshold one, no effect on the changes of the permeability loss rate should be observed, thus resulting in a constant (−∆*m*/∆*t*)* value. This value is the expected permeate reduction if Equation (3) holds, that is, at sub-threshold flux regimes, and must be compared to the measured one, hereafter reported as (−∆m/∆t)°. 

The application of Equation (6) implies the knowledge of the “a” parameter value: in this work, this value was calculated at the lowest available TMP value, where chances to work at sub-threshold operating conditions are highest.

Finally, by the application of the pressure cycling method, critical [Equation (7)] and threshold [Equation (8)] flux conditions are found at the lowest TMP value, where following conditions on the measured (−∆*m*/∆*t*)° values are met [33]:

(−∆*m*/∆*t*)° > 0 (7)

(−∆*m*/∆*t*)° > (−∆*m*/∆*t*)* (8)

The obtained results from the analysis were reported in Table 3.

**Table 3:** Determination of the critical and threshold flux for the nanofiltration (NF) membrane.

TMP (bar)	∆ *t* (h)	(−∆ *m*/∆*t*)° (L h^−2 ^m^−2 ^bar) × 10^−5^	(−∆ *m*/∆*t*)* (L h^−2 ^m^−2 ^bar) × 10^−5^
2	1	14.124	14.124
3	2	5.817	14.124
4	3	6.394	14.124
5	4	12.191	14.124
**6**	**5**	**16.130**	**14.124**
7	6	16.847	14.124

From the obtained results, no critical flux can be determined at the measured TMP values, since the relevant values of −∆*m*/∆*t*° are always higher than zero.

The determination of the “a” parameter in Equation (6) at 2 bar was successful, since the permeability decline stays within the measured limits, even at higher TMP values, thus confirming that the reference was taken at sub-threshold flux operating conditions. 

A threshold flux point exists of 6 bar, where (−∆*m*/∆*t*)° is starting to become higher than (−∆*m*/∆*t*)*, equal to 4.4 L h^−1^ m^−2^ and characterized by definition by a permeability loss of 14.124 × 10^−5^ L h^−2^ m^−2^ bar.

The permeate of NF has a final COD value of 102 mg L^−1^, corresponding to an overall rejection value of 95%. The permeate characteristics are reported in Table 4.

**Table 4:** Characteristics of the NF permeate.

Parameter	Unit	Value	Discharge limits
COD	mg/L	102	160
TSS	mg/L	0	80
NH_4_	mg/L	5.89	15
P	mg/L	<2.5	10
S	mg/L	0.09	1
Cr	mg/L	7.92	2

Although many parameters reach the requirements for discharge in surface waters, this is not the case of others, in particular, chromium and, possibly, other heavy metals. Therefore, RO must be applied to reach the targets of all parameters. 

The osmotic pressure of RO was equal to 9.71 bar and permeability equal to 0.364 L m^−2^ h^−1^ bar^−1^. The operating pressure relies only on economics. Since a capacity constraint exists on NF, given by the threshold flux, this latter aspect was taken into account, and as a consequence, a pressure of 22 bar is suggested for RO.

**Figure 2:**
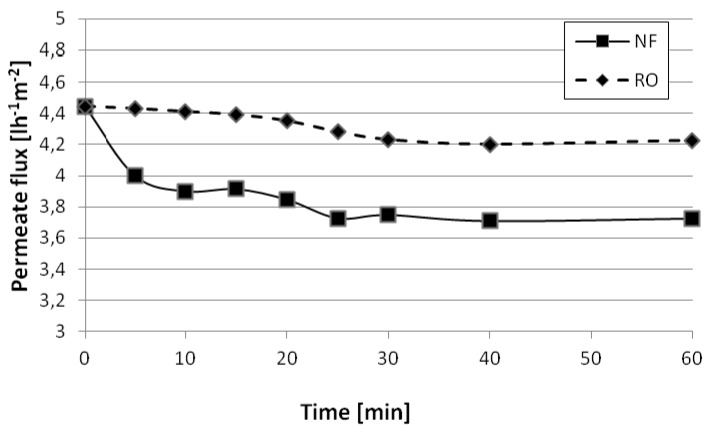
Profiles of the NF (full line) and reverse osmosis (RO) (dotted line) permeate fluxes during operation.

In Figure 2, the obtained permeate flux profiles, with a target of 95% of recovery, were reported at 6 bar and 22 bar for NF and RO, respectively. The characteristics of the obtained RO permeate stream is reported in Table 5. It is possible to notice that, besides fouling, an additional sensible permeate flux reduction exists, due to the operation in batch mode at a constant TMP value: the separation of the permeate stream leads to a solute concentration of the feedstock, and as a consequence, the membrane permeability decreases [25,26,31]. 

**Table 5:** Characteristics of the RO permeate.

Parameter	Unit	Value	Discharge limits
COD	mg/L	86	160
TSS	mg/L	0	80
Cr	mg/L	0.04	2

The experimental work showed the feasibility of performing the secondary treatment of the tannery wastewater effluents by membrane technology. In order to check the economic feasibility of the membrane process, a cost estimation of the proposed treatment was carried out, and the results were compared to the cost required by an external wastewater treatment service, relying on conventional biological processes. The proposed membrane plant, composed of NF and RO membrane processes in series, has the following characteristics, listed hereafter:

- **Investment costs**
  The membrane plant is designed for a capacity of 646 m^3^ h^−1^, in “feed & bleed” configuration, capable of guaranteeing the project permeate flux, equal to the one found at the end of NF operation and equal to 3.7 L m^−2^ h^−1^ (see Figure 2). In the case of NF, the permeate flux will reduce accordingly to the value of the “a” parameter, that is, of 3.7 L m^−2^ h^−1^ every 1,101 days. This means that NF membrane modules must be substituted every three years. This is not the case of RO, which is less prone to fouling, which may be substituted every five years. In the case of an overdesign of the surface area of NF of 100%, the number of required membrane modules, each having 32 m^2^ of membrane area, are 5456 and 2584 for NF and RO, respectively.
  Moreover, 4020 membrane housings, each capable of hosting two membrane modules, are needed. The hypothesized configuration adopted in this preliminary economic analysis was parallel, and the mean pressure drop estimated is approximately 1 bar. Moreover, the housings need to be served by pumps, piping and instrumentation (50% of the membrane housing costs), and in addition to this, the plant needs space and services (15% of total costs). Finally, a yearly fixed amortization rate, for 15 year plant lifetime, equal to 6%, is taken into account. The adopted costs for the membrane module and housing were 25 € m^−2^ and 400 €.
- **Operating costs**
  Mainly given by electricity consumption of the pumps and maintenance (10% of plant costs), a total of 80 kW of electric power is required. Costs are 0.11 € kWh^−1^.
- **Disposal of concentrates**
  The NF concentrates must be compressed by a filter press and then sent to disposal at fixed fees. The almost clear RO concentrate, rich in chromium, may be recycled back to the tannery process, in this study, without costs or benefits.

The application of membrane technology appears to be advantageous to the end user, that is, the tannery manufacturer, even if the economic benefit to recover chromium is not considered: the treatment and discharge of the wastewater stream is solved, with a minimum total cost savings of about 21%, if compared to the fixed fees of the external biological treatment plant service, offered at 2.28 € m^−3^ (see Table 6). The treatment process by membranes limits the disposal of concentrates to external services to 5%, permitting the discharge of 90% of the initial wastewater volume in surface waters and reusing 5% as chromium-rich concentrate at no cost. 

**Table 6:** Comparison of costs referred to 1 m^3^ of treated tannery wastewater stream in Italy.

Costs [€ m^−3^]	Membrane process
Amortization costs	1.44
Operating costs	0.12
Disposal of concentrates	0.24
TOTAL	1.80

Note: these costs are referring to those applied to the end user in Italy by external services.

## 4. Conclusions

A pretreated tannery wastewater stream was purified at pilot scale by membranes. After the determination of the threshold flux conditions for the NF membrane, found at 6 bar and equal to 4.4 L h^−1^ m^−2^, this latter value was taken into account during the design of the RO unit, which has not exhibited particular fouling issues and, thus, operated at 22 bar.

Membranes can be successfully used in substitution of other existing conventional secondary treatment plants, leading to a total cost savings equal to 21%.

## References

[1] Uberoi N.K. (2003). Environmental Management.

[2] Wiegant W.M., Kalker T.J.J., Sontakke V.N., Zwaag R.R. (1999). Full scale experience with tannery water management: An integrated approach. Water Sci. Technol..

[3] Sreeram K.J., Ramasami T. (2003). Sustaining tanning process through conservation, recovery and better utilization of chromium. Resour. Conserv. Recycl..

[4] Stoop M.L.M. (2003). Water management of production systems optimised by environmentally oriented integral chain management: Case study of leather manufacturing in developing countries. Technovation.

[5] Ahn D.H., Chung Y.C., Yoo Y.J., Pak D.W., Chang W.S. (1996). Improved treatment of tannery wastewater using zoogloea ranigera and its extracellular polymer in an activated sludge process. Biotechnol. Lett..

[6] Vijayaraghvan K., Murthy D.V.H. (1997). Effect of toxic substances inanaerobic treatment of tannery wastewater. Bioprocess Biosys. Eng..

[7] Wiemann M., Schenk H., Hegemann W. (1998). Anaerobic treatment oftannery wastewater with simultaneous sulphide elimination. Water Res..

[8] Di Iaconi C., Lopez A., Ramadori R., Passino R. (2003). Tannerywastewater treatment by sequencing batch biofilm reactor. Environ. Sci. Technol..

[9] Farabegoli G., Carucci A., Majone M., Rolle E. (2004). Biological treatment of tannery wastewater in the presence of chromium. J. Environ. Manag..

[10] Schrank S.G., Jos H.J., Moreira R.F.P.M., Schroder H.F. (2003). Fentons oxidation of various-based tanning materials. Desalination.

[11] Sekaran G., Chitra K., Mariappan K., Raghavan K.V. (1996). Removal of sulphide in anaerobically treated tannery wastewater by wet air oxidation. J. Environ. Sci. Health.

[12] Dogruel S., Ates G.E., Germirli B.F., Orhon D. (2004). Ozonation of nonbiodegradable organics in tannery wastewater. J. Environ. Sci. Health. A.

[13] Sacco O., Stoller M., Vaiano V., Ciambelli P., Chianese A., Sannino D. (2012). Photocatalytic Degradation of Organic Dyes under Visible Light on N-Doped Photocatalysts. Int. J. Photoenergy.

[14] Sannino D., Vaiano V., Sacco O., Ciambelli P. (2013). Mathematical modelling of photocatalytic degradation of methylene blue under visible light irradiation. J. Environ. Chem. Eng..

[15] De Caprariis B., di Rita M., Stoller M., Verdone N., Chianese A. (2012). Reaction-precipitation by a spinning disc reactor: Influence of hydrodynamics on nanoparticles production. Chem. Eng. Sci..

[16] Di Iaconi C., Lopez A., Ricco G., Ramadori R. (2001). Treatment options for tannery wastewater. I: Alkalinization with or without post-ozonation. J. Anal. Environ. Cultur. Herit. Chem..

[17] Orhon D., Sözen S., Çokgör E.U., Genceli E.A. (1998). The effect of chemical settling on the kinetics and design of activated sludge for tannery wastewaters. Water Sci. Technol..

[18] Song Z., Williams C.J., Edyvean R.G.J. (2004). Treatment of tannery wastewater by chemical coagulation. Desalination.

[19] Churchley J.H. (1994). Removal of sewage effluent—The use of a fullscaleozone plant. Water Sci. Technol..

[20] Stern S.R., Rodighiro I. (2003). Aerobic treatment of textile dyeing wastewater. Water Sci. Technol..

[21] Chu W. (2001). Dye removal from textile dye wastewaters using recycled alum sludge. Water Res..

[22] Field R.W., Wu D., Howell J.A., Gupta B.B. (1995). Critical flux concept for microfiltration fouling. J. Membr. Sci..

[23] Le-Clech P., Chen V., Fane T.A.G. (2006). Fouling in membrane bioreactors used in wastewater treatment. J. Membr. Sci..

[24] Field R.W., Pearce G.K. (2011). Critical, sustainable and threshold fluxes for membrane filtration with water industry applications. Adv. Colloid Interface Sci..

[25] Stoller M., Bravi M., Chianese A. (2013). Threshold flux measurements of a nanofiltration membrane module by critical flux data conversion. Desalination.

[26] Stoller M., Chianese A. (2006). Optimization of membrane batch processes by means of the critical flux theory. Desalination.

[27] Stoller M., Bravi M. (2010). Critical flux analyses on differently pretreated olive vegetation waste water streams: some case studies. Desalination.

[28] Stoller M. (2009). On the effect of flocculation as pretreatment process and particle size distribution for membrane fouling reduction. Desalination.

[29] Iaquinta M., Stoller M., Merli C. (2009). Optimization of a nanofiltration membrane process for tomato industry wastewater effluent treatment. Desalination.

[30] Stoller M. (2008). Technical optimization of a dual ultrafiltration and nanofiltration pilot plant in batch operation by means of the critical flux theory: A case study. Chem. Eng. Process..

[31] Stoller M. (2011). Effective fouling inhibition by critical flux based optimization methods on a NF membrane module for olive mill wastewater treatment. Chem. Eng. J..

[32] Ochando-Pulido J.M., Stoller M., Bravi M., Martinez-Ferez A., Chianese A. (2012). Batch membrane treatment of olive vegetation wastewater from two-phase olive oil production process by threshold flux based methods. Sep. Purif. Technol..

[33] Stoller M., de Caprariis B., Cicci A., Verdone N., Bravi M., Chianese A. (2013). About proper membrane process design affected by fouling by means of the analysis of measured threshold flux data. Sep. Purif. Technol..

[34] Stoller M., Ochando Pulido J.M., Chianese A. (2013). Comparison of Critical and Threshold Fluxes on Ultrafiltration and Nanofiltration by Treating 2-phase or 3-phase Olive Mill Wastewater. Chem. Eng. Trans..

[35] Stoller M. (2013). A Three Year Long Experience of Effective Fouling Inhibition by Threshold Flux Based Optimization Methods on a NF Membrane Module for Olive Mill Wastewater Treatment. Chem. Eng. Trans..

